# Is It Possible to Predict Difficulties During Laparoscopic Sleeve Gastrectomy? A Single Centre Experience

**DOI:** 10.3390/jpm14111098

**Published:** 2024-11-08

**Authors:** Magdalena Pisarska-Adamczyk, Tomasz Stefura, Piotr Małczak, Piotr Major, Michał Wysocki

**Affiliations:** 1 Department of Medical Education, Jagiellonian University Medical College, 31-008 Kraków, Poland; tomasz.stefura@uj.edu.pl; 2 2nd Department of General Surgery, Jagiellonian University Medical College, 30-688 Kraków, Poland; piotr.malczak@uj.edu.pl (P.M.); piotr.major@uj.edu.pl (P.M.); 3 Department of General Surgery and Surgical Oncology, Ludwik Rydygier Memorial Hospital in Cracow, 31-820 Kraków, Poland; michal92wysocki@gmail.com

**Keywords:** sleeve gastrectomy, intraoperative difficulties, risk factors

## Abstract

Introduction: Laparoscopic sleeve gastrectomy (LSG) is a widely performed bariatric surgery across the globe. Understanding preoperative risk factors for possible intraoperative complications can aid in predicting surgical outcomes and shaping the approach to the procedure. This study aimed to identify and analyze potential risk factors associated with intraoperative difficulties during LSG. Patients and methods: The analysis encompassed consecutive patients who underwent LSG from 2017 to 2020. Patients who encountered intraoperative difficulties during the procedure were categorized into Group 1, whereas those who did not experience such complications were placed in Group 2. To identify potential risk factors for intraoperative challenges, a thorough evaluation of demographic characteristics was conducted, including variables such as age, body mass index (BMI), comorbidities, and previous surgical history. Results: Group 1 included 37 patients (11.71%), while Group 2 comprised 279 patients (88.29%). Apart from higher rates of diabetes, pulmonary disease, and sleep apnea in Group 1, no significant differences were observed between the groups regarding demographic parameters. A univariate logistic regression analysis identified several risk factors associated with intraoperative difficulties, including a body mass index (BMI) greater than 50 kg/m^2^ (OR 2.15, 95%, CI 1.05–4.39, *p* = 0.0362), the experience of the operating surgeon (OR 9.22, 95% CI 4.31–19.72, *p* = 0.0058), the presence of diabetes (OR 2.44, 95% CI 1.19–4.98, *p* = 0.0146), and pulmonary disease (OR 12.22, 95% CI 1.97–75.75, *p* < 0.0001). In multivariate logistic regression analysis, only the surgeon’s experience (OR 8.61, 95% CI 3.75–19.72, *p* < 0.0001) remained a significant factor influencing intraoperative difficulties. Conclusions: The sole significant factor influencing the occurrence of intraoperative difficulties was the level of the surgeon’s experience

## 1. Introduction

The anticipation of intraoperative difficulties influences the decisions regarding the operative approach, leading to more individualized treatment for high-risk patients and improving postoperative outcomes. By proactively identifying and addressing potential challenges, surgeons can tailor their techniques and strategies to each patient’s specific needs, thereby enhancing safety, reducing complications, and promoting a smoother recovery process. [1,2].

Laparoscopic sleeve gastrectomy (LSG) and Laparoscopic Roux-en-Y Gastric Bypass (LRYGB) are currently among the most commonly performed bariatric procedures both globally and in Poland [3,4]. LSG, in particular, is often regarded as a less technically demanding operation compared to LRYGB, largely due to its simpler surgical approach and shorter operative time. This relative ease of performance frequently positions LSG as the preferred initial bariatric procedure for surgical residents who are still acquiring expertise in advanced minimally invasive techniques [5,6]. Despite its perceived simplicity, LSG has demonstrated the ability to produce substantial bariatric and metabolic benefits, including significant and sustained weight loss, as well as marked improvements in obesity-related comorbidities such as type 2 diabetes, hypertension, and dyslipidemia [7,8,9,10]. The growing popularity of LSG as a bariatric option reflects its balance between procedural efficiency and favorable long-term outcomes, making it a critical component in the management of obesity and associated metabolic disorders.

Bariatric surgery is generally associated with a low risk of both morbidity and mortality, making it a relatively safe intervention for weight loss. However, certain technical challenges that arise during laparoscopic sleeve gastrectomy (LSG) can contribute to postoperative complications, highlighting the importance of managing these difficulties effectively [11]. Understanding the impact of intraoperative challenges on postoperative outcomes, as well as identifying the factors that increase their likelihood, is crucial for developing strategies to mitigate them and for selecting appropriate candidates for LSG [12]. It is important to recognize, however, that not all intraoperative difficulties, even the most severe ones, lead to adverse outcomes for the patient. Anticipating the potential for complications allows for the allocation of highly experienced surgical teams during the procedure, enhancing the overall safety and success of the surgery. By improving our ability to foresee and manage these challenges, we can further minimize risks and optimize patient outcomes in bariatric surgery.

The aim of this study was to identify potential risk factors that contribute to intraoperative difficulties during laparoscopic sleeve gastrectomy (LSG). By investigating various patient characteristics and surgical variables, the study seeks to pinpoint factors that increase the likelihood of complications.

## 2. Materials and Methods

### 2.1. Study Design

**Figure 1:**
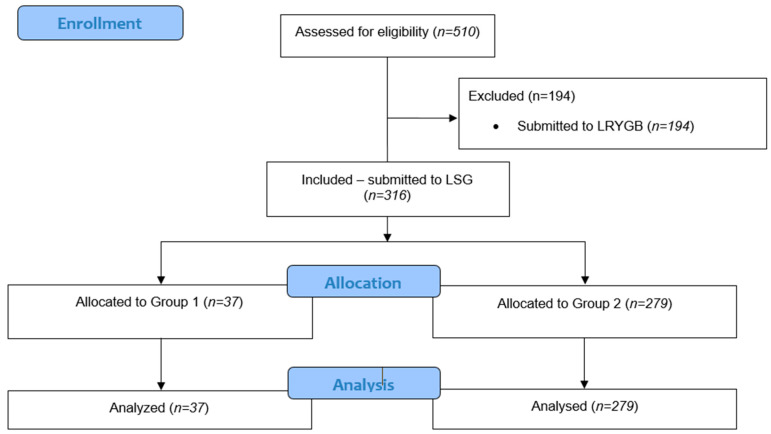
Flowchart.

The study was a prospective observational analysis of consecutive patients undergoing laparoscopic sleeve gastrectomy (LSG) from 2017 to 2020 at a tertiary referral university hospital. It encompassed all patients over the age of 18 who met the eligibility criteria for bariatric surgery as recommended by the Metabolic and Bariatric Surgery Section of the Polish Surgical Society. These criteria included a body mass index (BMI) greater than 40 kg/m^2^ or a BMI exceeding 35 kg/m^2^ in the presence of obesity-related comorbidities such as type 2 diabetes, hypertension, obstructive sleep apnea, and lipid disorders [13,14,15]. Patients who had undergone Laparoscopic Roux-en-Y Gastric Bypass (LRYGB) or were undergoing revision surgery were excluded from the analysis. The flow of patients through the study is illustrated in Figure 1.

Data collection was performed by the authors, who were also directly involved in the treatment process. Database included demographic and operative characteristics (sex, age, max. preoperative BMI, preoperative weight loss, ASA (American Society of Anesthesiologists) scale, the presence of preoperative comorbidities, cardiovascular disease, hypertension, diabetes, pulmonary disease, metabolic syndrome, experience of the operating surgeon, and experience of anesthesiologist).

The procedure was considered difficult when the operative time was over 200 min, blood loss was over 200 mL, it was necessary to perform the conversion, the number of staplers used during the operation exceeded 5, or when there were any technical difficulties in using staplers (for example, when a stapler cut but did not suture tissue) [16]. Data were collected using a standardized checklist, which was completed by the lead surgeon and a surgical assistant to ensure consistency.

Patients with intraoperative difficulties were assigned to Group 1, and patients without intraoperative difficulties to Group 2. An experienced surgeon was defined as an operator proficient in advanced laparoscopic operations (gastrointestinal oncological surgery, hernia repair, or LRYGB) who also performed LSG at least 100 times [17]. The incidence of postoperative complications was assessed in accordance with the Clavien–Dindo classification [18], and the LOS (length of stay) was defined as the duration of an inpatient episode of care, measured from the day of admission to the day of discharge, and calculated based on the number of nights spent in the hospital. Readmission was defined as any patient hospitalization related to the surgery within 30 days after being discharged home.

### 2.2. Surgical Technique

The surgical method for LSG was consistently applied across all patients. The description of the operative technique was presented in one of our earlier papers [19].

The surgical approach for laparoscopic sleeve gastrectomy has been standardized. Typically, four trocars are inserted to facilitate the procedure. Tissue dissection and coagulation are performed using either a sealer/divider or ultrasonic shears, such as LigaSure Atlas™ (Covidien, Dublin, Ireland) or SonoSurg™ (Olympus, Hamburg, Germany). To shape the gastric sleeve, a 36-French bougie is placed along the lesser curvature inside the stomach. Gastrectomy begins 4–5 cm above the pylorus, with a continuous application of linear staplers directed towards the angle of His. The stapler line is then reinforced with a continuous 3–0 PDS stitch and, in later years, with a V-Lock stitch for additional security. Finally, standard closure of the 10/12 mm port sites is performed to reduce the risk of herniation.

In all patients, perioperative care followed the principles of Enhanced Recovery After Surgery protocol according to ERAS Society Guidelines [20,21].

### 2.3. Statistical Analysis

All data were prospectively gathered and entered into a computerized database. Statistical analyses were conducted using Statsoft STATISTICA v.13. The results are presented as mean ± standard deviation (SD), median and interquartile range (IQR), and odds ratio (OR) with 95% confidence intervals (CIs) where applicable. The choice of statistical tests was based on the nature of the variables. Chi-square tests were employed for categorical variables. The Shapiro–Wilk test was used to assess the normality of the data distribution. For normally distributed quantitative data, Student’s *t*-test was utilized. For data that were not normally distributed, the Mann–Whitney U test was applied. Logistic regression was performed to assess potential risk factors affecting intraoperative difficulties. We analyzed the influence of the following factors on intraoperative difficulty: sex; age; max. preoperative BMI; preoperative weight loss; ASA (American Society of Anesthesiologists) scale; the presence of preoperative comorbidities; cardiovascular disease; hypertension; diabetes; pulmonary disease; metabolic syndrome; experience of the operating surgeon; and experience of anesthesiologist. Finally, the variables in the univariate logistic regression analysis that had a *p*-value < 0.1 based on univariate analysis were used to build a multivariate logistic regression model. Results were regarded as statistically significant if the *p*-value was below 0.05.

### 2.4. Ethical Considerations

All procedures were conducted in accordance with the ethical standards established by the 1964 Declaration of Helsinki and its subsequent amendments (Fortaleza). Written informed consent was obtained from all patients prior to surgery. The study received approval from the local Ethics Review Committee (1072.6120.225.2017).

## 3. Results

Out of 316 LSGs, the procedure was considered difficult in 37 cases. The main reason for this assessment was the operative time exceeding 200 min in six patients (16.2%) due to technical obstacles associated with laparoscopy. LSG in four patients (1.27%) was associated with blood loss above 200 mL. Injured vessels were sealed by an electrothermal bipolar instrument. The number of used staplers during the procedure was over five in 29 cases (9.18%). Additionally, in 1 patient, conversion was needed.

No statistically significant differences between groups were observed for demographic parameters such as age, sex, BMI on the day of operation, maximal preoperative BMI, preoperative weight loss, physical status assessed according to the American Society of Anesthesiologists (ASA) classification, and the incidence of any comorbidity, including a number of cardiovascular diseases, hypertension, and steatohepatitis. The rates of diabetes, pulmonary disease, or obstructive sleep apnea were significantly higher in Group 1 compared to Group 2 (Table 1).

There were statistically significant differences in the mean operative time between the two groups, with Group 1 experiencing a mean time of 142.8 ± 51.8 min compared to 103.2 ± 36.6 min in Group 2 (*p* < 0.05). This indicates that procedures in Group 1 took significantly longer on average. Additionally, the mean intraoperative blood loss was substantially greater in Group 1, with an average of 240 ± 31.1 mL compared to 43 ± 30.8 mL in Group 2 (*p* < 0.05). Moreover, patients in Group 1 required more staplers during the procedure, with an average of 5.9 ± 0.9 compared to 4.2 ± 0.6 in Group 2 (*p* < 0.05). Only one patient from the analyzed group required conversion for the classic procedure. Detailed information about the operative parameters of the analyzed groups is presented in Table 2.

Univariate logistic regression analysis identified several factors associated with intraoperative difficulties, including a body mass index (BMI) greater than 50 kg/m^2^ (odds ratio [OR] 2.15, 95% confidence interval [CI] 1.05–4.39, *p* = 0.0362), the experience level of the operating surgeon (OR 9.22, 95% CI 4.31–19.72, *p* = 0.0058), and the presence of certain comorbidities such as diabetes (OR 2.44, 95% CI 1.19–4.98, *p* = 0.0146) or pulmonary disease (OR 12.22, 95% CI 1.97–75.75, *p* < 0.0001) (Table 3). However, multivariate logistic regression analysis revealed that, among these factors, only the lack of experience of the operating surgeon remained significantly associated with an increased risk of intraoperative difficulties (OR 8.60, 95% CI 3.75–19.72, *p* < 0.0001), as detailed in Table 3.

Table 1 shows that the complication rate in Group 1 was 21.62% compared to 6.45% in Group 2 (*p* < 0.0001). However, when evaluating the severity of complications using the Clavien–Dindo classification, no significant differences were found between the two groups (*p* = 0.0794). Additionally, the median length of stay (LOS) was notably longer in Group 1, with a median of 5 days compared to 3 days in Group 2 (*p* < 0.001). Furthermore, patients in Group 1 who encountered intraoperative difficulties were significantly more likely to experience unplanned readmissions after discharge, with a rate of 8.11% versus 2.15% in Group 2 (*p* = 0.0407). The predominant reasons for these readmissions were postoperative dehydration and electrolyte imbalances. Detailed information is provided in Table 4.

## 4. Discussion

Our study showed that 12% of LSG operations were associated with intraoperative difficulties, which significantly influenced the further course of treatment. The incidence of difficulty during operation resulted in higher rates of postoperative complications, longer time of hospitalization, and higher readmission rates. Our results revealed that the only unrelated risk factor affecting intraoperative difficulties was operations performed by a less experienced surgeon.

Preoperative knowledge concerning the incidence and risk factors for potential intraoperative difficulties may help to predict the outcomes of the operation [22,23]. For instance, inflammation induced by preoperative endoscopic retrograde cholangiopancreatography significantly increases the difficulty of laparoscopic cholecystectomy resulting in a higher conversion rate [24]. Chemoradiotherapy prior to surgical treatment of patients with rectal cancer is also associated with increased surgical difficulty, which is reflected in a longer operative time [25]. We will try to demonstrate a similar relationship in the group of patients undergoing bariatric procedures.

The reported incidence of intraoperative difficulties among other authors ranges between 3.3% and 14% [26,27,28]. In our study, the most common intraoperative difficulty was the number of staplers required during surgery exceeding five, followed by an operative time of over 200 min. Data published by Kaska et al. presents a different approach to the subject, where the restrictive operative area, bleeding, and calibration tube entrapment were mentioned as the most common intraoperative difficulties associated with LSG [28]. A study published in 2007 by Braghetto et al. described the treatment course of patients submitted to LSG, where 14% of operations were associated with intraoperative difficulties such as intraoperative leakage, bleedings, and the fixation of the nasogastric tube in the mechanical suture line [24]. The intraoperative difficulties during LSG mentioned by Rubin et al. also include intraoperative leakage, bleeding, and the fixation of the temperature sensor probe in the staple line [27].

The only factor responsible for the incidence of intraoperative difficulties in our study is the experience of the surgeon. Every surgeon in training is overcoming the learning curve. For each type of surgery, the number of procedures necessary to carry out this learning curve is different. Some authors reported that proficiency in performing laparoscopic sleeve gastrectomy required 68 cases, whereas others believed that at least 100 procedures are necessary to achieve proper experience [17]. Overcoming the learning curve is not only associated with a greater number of intra- but also postoperative complications. In our study, when LSG was performed by a less experienced surgeon, the odds of intraoperative difficulties increased almost nine times (OR 8.61). The intraoperative difficulties associated with the operator’s less experience were mainly limited to the significantly extended duration of the procedure. And most importantly, these difficulties were not associated with the occurrence of postoperative complications.

Although numerous studies have shown that a higher BMI increases mortality and complication rates, in our work, a maximal preoperative BMI over 50 kg/m^2^ did not contribute to the incidence of intraoperative difficulties [29,30]. Benedix et al. showed that the BMI was associated with increased leaks and was the highest for patients with a preoperative BMI between 50 and 59.9 kg/m^2^ [31]. Also, according to Buchwald et al., a BMI > 50 kg/m^2^ besides male sex, age > 65 years, and obstructive sleep apnea are well-established risk factors for complications and mortality [32]. Aurora et al., in a systematic analysis of 4888 patients, showed that in the group of patients with a BMI > 50 kg/m^2^, the incidence of leaks was higher, but the difference was not statistically significant [33]. Similarly, Major et al. showed that a higher BMI increased the complication rate only for LRYGB, but did not increase this score in patients with LSG [34]. The negative impact of BMI on leakage is also shown by other authors [35,36]. However, the results presented are difficult to compare to ours due to the cut-off point being determined differently.

We have not shown that other demographic or operative factors are associated with a greater risk of intraoperative difficulties. As mentioned in other authors’ work, factors such as age, sex, or some comorbidities in our material did not prove to be significant factors. This may be due to differences in perioperative care. The ERAS protocol is currently part of our routine perioperative care in bariatric surgery. All patients in our study were treated in accordance with the principles of the multimodal pathway, which has been shown to accelerate recovery, reduce the incidence of complications, and shorten LOS. Studies in different patient groups treated with the ERAS protocol have shown that many of the traditional demographic factors have little or no impact on short-term outcomes [37,38,39]. Similar conclusions about intraoperative difficulties can be drawn from our work.

Our study has several limitations inherent to its single-center design. Intraoperative difficulties were observed in only approximately 12% of patients, resulting in a relatively small sample size for analysis. Additionally, the study is limited by the lack of long-term outcome analysis, as it focuses solely on short-term results. Furthermore, we did not account for the potential effects of the learning curve throughout the study period, which may influence the incidence of intraoperative difficulties during laparoscopic sleeve gastrectomy (LSG). Future studies with larger, multi-center cohorts, a focus on long-term outcomes, and a consideration of the surgical learning curve are needed to validate these findings and further elucidate factors impacting LSG outcomes.

## 5. Conclusions

Even when no complications were noted after LSG and the postoperative period was standard, as many as 12% of patients considered the operation difficult. The primary determinant influencing the occurrence of intraoperative complications within our cohort was the surgeon’s level of expertise. Experienced surgeons were less likely to encounter technical difficulties. Notably, patient-specific factors, including age, body mass index, and pre-existing health conditions, did not have a measurable impact on intraoperative outcomes in our cohort. These findings underscore the importance of surgical proficiency and training in achieving optimal procedural outcomes.

## Figures and Tables

**Table 1:** Demographic and operative parameters of studied group.

Parameter	Value	Group 1	Group 2	*p* Value
Number of patients, *n* (%)	316 (100%)	37 (11.71%)	279 (88.29%)	-
Females, *n* (%)	208 (65.8%)	21 (56.76%)	187 (67.03%)	0.2159
Males, *n* (%)	108 (34.2%)	16 (43.24%)	92 (32.97%)	
Mean age, years ± SD	40.8 ± 11.1	42.8 ± 11.8	40.5 ± 11	0.2444
Mean BMI on a day of operation, kg/m^2^ ± SD	45.7 ± 6.2	47.3 ± 6	45.5 ± 6.3	0.1069
Mean maximal preoperative BMI, kg/m^2^ ± SD	47.4 ± 6.5	48.9 ± 6.6	47.2 ± 6.5	0.1226
Mean preoperative weight loss, kg ± SD	5.1 ± 7.9	6.2 ± 12	4.94 ± 7.4	0.4194
ASA 1, *n* (%)	16 (5.1%)	3 (8.11%)	13 (4.66%)	0.6213
ASA 2, *n* (%)	237 (75.0%)	26 (70.27%)	211 (75.63%)	
ASA 3, *n* (%)	63 (19.9%)	8 (21.62%)	55 (19.71%)	
Any comorbidity, *n* (%)	240 (75.9%)	32 (86.49%)	208 (74.55%)	0.1105
Cardiovascular, *n* (%)	46 (14.6%)	7 (18.92%)	39 (13.98%)	0.4233
Hypertension, *n* (%)	202 (63.92%)	27 (72.97%)	175 (62.72%)	0.2324
Diabetes, *n* (%)	76 (24.1%)	15 (40.54%)	61 (21.86%)	0.0125
Pulmonary disease, *n* (%)	5 (1.6%)	3 (8.11%)	2 (0.72%)	<0.0001
Obstructive sleep apnea, *n* (%)	29 (9.2%)	7 (18.92%)	22 (7.89%)	0.0289
Steatohepatitis, *n* (%)	180 (57.0%)	23 (62.16%)	157 (56.27%)	0.5265
Metabolic syndrome, *n* (%)	168 (53.16%)	25(67.57%)	143 (51.25%)	0.0620

**Table 2:** Operative parameters of analyzed groups.

Parameter	Value	Group 1	Group 2	*p* Value
Mean operative time, min ± SD	107.9 ± 40.7	142.8 ± 51.8	103.2 ± 36.6	<0.0001
Mean intraoperative blood loss, ml ± SD	60 ± 30.9	240 ± 31.1	43 ± 30.8	<0.0001
Median number of staplers (IQR)	4 (4–5)	5.9 ± 0.9	4.2 ± 0.6	<0.0001
Conversion, *n* (%)	1 (0.3%)	1 (2.7%)	-	-

**Table 3:** Logistic regression affecting intraoperative difficulties.

	Univariate Logistic Regression			Multivariate Logistic Regression		
Parameter	OR	95% CI	*p* Value	OR	95% CI	*p* Value
Sex (female vs. male)	0.64	0.32–1.3	0.6457			
Age (≥40 vs. <40)	1.44	0.72–2.88	0.3094			
Max. preoperative BMI (≥50 vs. <50 kg/m^2^)	2.10	0.98–4.5	0.0570	1.03	0.23–4.69	0.9715
Preoperative weight loss (≥5 vs. <5 kg)	0.61	0.29–1.31	0.2110			
ASA (III vs. I–II)	1.12	0.49–2.59	0.7849			
Any comorbidity (yes vs. no)	2.18	0.82–5.82	0.1182			
Cardiovascular disease (yes vs. no)	1.44	0.59–3.5	0.4254			
Hypertension (yes vs. no)	1.59	0.74–3.42	0.2355			
Diabetes (yes vs. no)	2.44	1.19–4.98	0.0146	2.08	0.79–5.43	0.1325
Pulmonary disease (yes vs. no)	12.22	1.97–75.75	<0.0001	7.02	0.89–40.8	0.0954
Metabolic syndrome (yes vs. no)	1.98	0.96–4.1	0.0654	1.03	0.34–3.02	0.9599
Surgeon (experienced vs. novice)	9.22	4.31–19.72	<0.0001	8.60	3.75–19.72	<0.0001

**Table 4:** Postoperative outcomes.

Parameter	Group 1	Group 2	*p* Value
Patients with complications, *n* (%)	21.62%	6.45%	*p* < 0.0001
Median LOS, days (IQR)	5 (4–6)	3 (2–3)	*p* < 0.001
Reasmission, *n* (%)	8.11%	2.15%	*p* = 0.0407

## Data Availability

Data without personal patient information can be available upon request via email to the main author.
